# Cost-Effectiveness of Surgery Versus Functional Bracing for Humeral Shaft Fractures in Adults: A Prespecified Economic Evaluation of the Finnish Shaft of the Humerus (FISH) Trial

**DOI:** 10.2106/JBJS.25.00867

**Published:** 2026-02-24

**Authors:** Cyrill Suter, Thomas Ibounig, Aleksi Reito, Henrik Mattila, Bakir O. Sumrein, Antti P. Launonen, Mika Paavola, Teppo L.N. Järvinen, Simo Taimela, Lasse Rämö

**Affiliations:** 1 Finnish Centre for Evidence-Based Orthopaedics (FICEBO), Department of Orthopaedics and Traumatology, Helsinki University Hospital, University of Helsinki, Helsinki, Finland; 2 Department of Orthopaedics and Traumatology, Tampere University Hospital, University of Tampere, Wellbeing Services County of Pirkanmaa, Tampere, Finland

## Abstract

**Background:**

Humeral shaft fractures commonly affect working-age adults and can lead to prolonged work absence and substantial economic burden. Although surgical fixation and functional bracing offer comparable functional outcomes, their relative cost-effectiveness remains unclear.

**Methods:**

We conducted a prespecified economic evaluation alongside a multicenter, superiority, randomized clinical trial at 2 Finnish university hospitals between 2012 and 2018. Eighty-two adults (mean age, 48.9 years; 38 women) with displaced, closed humeral shaft fractures were randomly assigned to surgical fixation (n = 38) or functional bracing (n = 44) and followed for 2 years. The primary outcome was the incremental net monetary benefit (INMB) based on quality-adjusted life years (QALYs) measured with the 15-dimensional (15D) instrument, analyzed from both societal and health-care perspectives.

**Results:**

From a societal perspective, surgical treatment was both more effective and less costly than bracing. The mean total cost per patient was €23,680 for surgery and €30,389 for bracing, yielding an INMB of €9,423 (95% confidence interval [CI], €4,139 to €14,609). Cost-effectiveness acceptability curves showed that surgery was highly likely to be cost-effective across all willingness-to-pay thresholds up to €120,000 per QALY. The cumulative QALYs from 6 weeks to 2 years post-injury were 1.776 (95% CI, 1.725 to 1.827) for surgery and 1.705 (95% CI, 1.641 to 1.769) for bracing, corresponding to a QALY difference of 0.071 (95% CI, 0.012 to 0.130) in favor of surgery. From the health-care perspective, functional bracing was less costly (€4,904 versus €10,967) and therefore more cost-effective, with an INMB of −€4,087 (95% CI, −€5,215 to −€3,054). When considering only direct medical costs, surgery was unlikely to be cost-effective at thresholds below €80,000 per QALY, reaching a 75% probability of cost-effectiveness only at €120,000 per QALY.

**Conclusions:**

Surgery is cost-effective when societal costs are considered. Functional bracing remains a reasonable option, particularly for patients less affected by time away from work. Shared decision-making should incorporate both economic and individual patient factors.

**Level of Evidence:**

Economic Level I. See Instructions for Authors for a complete description of levels of evidence.

Humeral shaft fractures represent 1% to 3% of all adult fractures^1,2^. In the United States, these fractures are projected to result in around 76,000 emergency department visits by 2030, assuming that the age-adjusted incidence rates remain constant^3^. In younger individuals, humeral shaft fractures carry a substantial societal burden by impacting workforce participation and productivity. This burden highlights the importance of selecting treatments that facilitate a timely return to work and to other daily activities.

Choosing the ideal treatment for patients with humeral shaft fractures requires careful weighing of the distinct benefits and risks associated with surgical and nonsurgical management. Functional bracing avoids operative complications and typically yields good functional outcomes when fracture healing is successful; however, it carries a notable risk of nonunion (up to 33%), with the subsequent need for secondary surgery and a prolonged recovery^4-6^. Conversely, surgery offers advantages, including earlier mobilization and lower rates of nonunion (0% to 9%)^7,8^, but introduces inherent surgical risks, such as iatrogenic radial nerve palsy (4% to 9%) and infection (1% to 5%)^9-13^. In previous studies using simulations or retrospective analyses to perform economic evaluations of treatments for humeral shaft fractures, primary surgery was favored over nonoperative treatment, particularly when productivity losses were included^14-16^.

We previously published clinical outcomes from the Finnish Shaft of the Humerus (FISH) randomized clinical trial (RCT), which showed comparable functional results between surgery and functional bracing^17-19^. Building on these findings, the present study tested the hypothesis that, despite higher upfront health-care costs, surgical treatment would prove more cost-effective than functional bracing when productivity losses are considered. Our goal was to provide the first trial-based cost-effectiveness comparison of these treatments in order to inform clinical and policy decisions regarding the management of humeral shaft fractures.

## Materials and Methods

The FISH trial (ClinicalTrials.gov NCT01719887) was a multicenter, superiority RCT conducted at Helsinki University Hospital and Tampere University Hospital from 2012 to 2018. Ethics approval was obtained. The protocol was previously published^20^.

To recapitulate in brief: adults with unilateral, displaced, closed humeral shaft fractures were randomized to surgery (open reduction and plate fixation) or functional bracing (hereafter referred to as bracing). The exclusion criteria and treatment protocols are detailed in eAppendix 1. Race and ethnicity data were not collected, as those variables were not included in the original study design.

### Costs and Outcomes

This analysis followed the Consolidated Health Economic Evaluation Reporting Standards^21^.

#### Direct Medical Costs

Direct medical costs included expenses for hospital admissions, diagnostics, surgery, primary care visits, follow-up appointments, rehabilitation, and medications. Cost data were sourced from the Hospital District of Helsinki and Uusimaa pricing system.

Surgical procedures were classified as either primary or secondary. Primary surgeries (i.e., surgeries performed within 2 weeks of the injury) were costed higher than secondary surgeries (i.e., delayed surgeries). Services delivered by primary care clinics or private providers were not included in the hospital district’s pricing list. Instead, primary care and private service costs were estimated from national databases (see Appendix eTable 1)^22,23^.

Drug costs were obtained from Finland’s centralized electronic prescription system, which is maintained by the Social Insurance Institution of Finland (Kela). This system digitally records all prescriptions nationwide, providing real-time access for all pharmacies.

#### Indirect Costs

Indirect costs were assessed by estimating productivity losses due to work absence. Work absence days (WADs) were determined on a case-by-case basis with use of data from the centralized work absence registry of Finland (Kela) and sick-leave certificates from hospital records to ensure patient-level accuracy.

Productivity losses were calculated by multiplying WADs by daily cost estimates. The primary analysis used direct employer costs (the average base salary plus a 21% overhead), resulting in a daily estimate of €222^24^. The supplementary analysis adopted a broader economic perspective, applying the Confederation of Finnish Industries’ 2021 estimate of €370 per WAD, which accounts for employer friction costs and macroeconomic factors (see eAppendix 2 and Appendix eFig. 1). For students (3 in each treatment group), the primary analysis applied the Kela minimum daily sickness allowance of €32. Additional indirect costs included home care assistance covered by publicly funded social services.

Cost data were collected via patient questionnaires at the baseline visit and at follow-up visits from 6 weeks to 2 years after the injury and supplemented with centralized health-care data and hospital records for completeness. For patients lost to follow-up, costs were included up to the point of the patient’s discontinuation. However, prescriptions and reimbursed work absence data throughout the follow-up period were obtained for all participants through Kela.

All costs were adjusted to 2023 price levels (current exchange rate: €1 = $1.17) with use of the Finnish Consumer Price Index^24^.

#### Effectiveness Measure

The effectiveness measure in our cost-effectiveness analysis (CEA) was the between-group difference in quality-adjusted life years (QALYs) using the 15-dimensional (15D) instrument. The 15D score ranges from 0 (death) to 1 (full health) and has a minimal important difference of 0.03^25,26^. Given the sharp decline in quality of life immediately after the injury and the high variability during the early recovery phase, the first 6 weeks after the injury were excluded from the analysis. Therefore, QALYs were estimated using a time-weighted average of the 15D scores starting from 6 weeks post-injury.

### Cost-Effectiveness Analysis

We evaluated the cost-effectiveness of surgical treatment versus bracing with use of the incremental net monetary benefit (INMB) as the primary metric. The INMB converts health gains into monetary terms using a willingness-to-pay (WTP) threshold and then subtracts incremental costs, providing a single value that is positive if the treatment is cost-effective. The base-case analysis adopted a societal perspective, incorporating both direct health-care costs and productivity losses over a 2-year follow-up period. A health-care perspective, limited to direct treatment costs, was also evaluated for comparison. In addition to the INMB, we calculated incremental cost-effectiveness ratios (ICERs), which represent the extra cost required to gain 1 additional unit of health (e.g., €/QALY) when comparing 2 treatments (see eAppendix 3).

A within-trial stochastic CEA was conducted over the 2-year period. Treatments were coded as A (surgery) or B (bracing), with the INMB calculated as follows: INMB = incremental effects × λ − incremental costs, where λ is the WTP threshold per QALY gained. Although Finland lacks formal WTP thresholds, we used a WTP threshold of €35,000 per QALY on the basis of prior studies and National Institute for Health and Care Excellence thresholds (£20,000 to £30,000 per QALY)^27^. Incremental effects and costs were estimated as group means with use of seemingly unrelated regression (SUR) via the fitsur function in the systemfit package^36^ of R (2023; R Foundation for Statistical Computing), accounting for the correlation between costs and QALYs. Models were adjusted for treatment arm, age, study center, baseline quality of life, physical workload, and smoking status.

Costs and 15D scores beyond year 1 were discounted at a 3% annual rate^28^. Missing data were handled via multiple imputation, using all covariates except treatment group. Weighted means and the SUR were calculated after imputation. To assess uncertainty, we generated 95% confidence intervals (CIs) for costs, effects, and the INMB with use of nonparametric bootstrapping with 5,000 replications, resampling with replacement from the original data set^29^. Multiple imputed data sets (5 in total) were passed to the bootstrapping function, and pooled coefficients from these data sets were used as outputs for each replication.

Results are presented with cost-effectiveness planes and cost-effectiveness acceptability curves (CEACs). The cost-effectiveness planes display the distribution of bootstrapped incremental costs and QALYs, whereas the CEACs show the probability that surgery is cost-effective across WTP thresholds from €0 to €120,000^30^. All analyses were conducted in RStudio (version 4.3.2; Posit Software).

### Sensitivity Analysis

To evaluate the robustness of the findings, we conducted 1-way sensitivity analyses. Hospital costs and productivity losses were varied independently by ±50% to assess their individual impact. To address potential outlier bias, we applied winsorization, adjusting extreme cost values to the 10th and 90th percentiles. This procedure was conducted in 2 separate analyses: 1 for total costs and another for productivity losses.

## Results

**Fig. 1:**
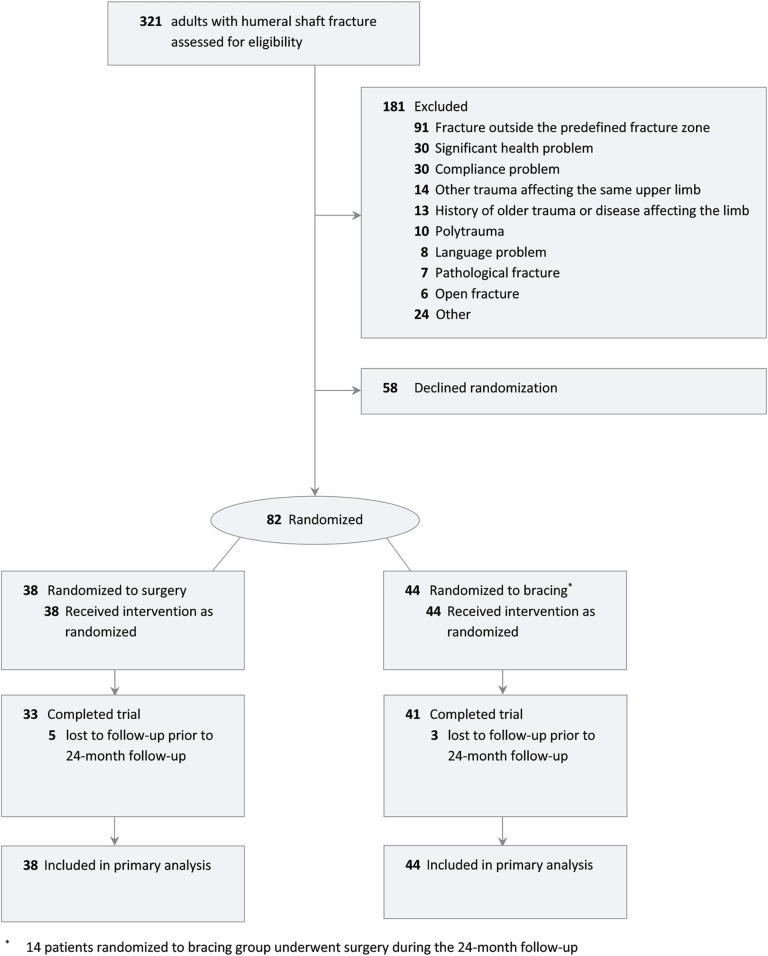
Flowchart of the study. Patients with >1 reason for exclusion are counted more than once.

Of 321 patients screened, 82 (mean age, 48.9 years; 38 women) were randomized: 38 to surgery and 44 to bracing. At 2 years, 74 patients (90%) completed follow-up (Fig. 1); 5 patients in the surgery group and 3 in the bracing group were lost to follow-up. Baseline characteristics were similar between the groups (Table 1). All patients in the surgery group and 68% of the patients in the bracing group achieved healing; 32% of the bracing group required conversion to primary or secondary surgery (see Appendix eTable 2).

**Table 1:** Baseline Demographics and Clinical Characteristics*

Characteristic	Surgery Group (N = 38)	Functional Bracing Group (N = 44)
Age at allocation *(yr)*	49.6 ± 18.2 (19-81)	48.4 ± 16.2 (19-80)
Women *(no. of patients)*	18 (47%)	20 (45%)
Men *(no. of patients)*	20 (53%)	24 (55%)
Weight *(kg)*	84 ± 21	85 ± 16
Height *(cm)*	173 ± 9	174 ± 9
Body mass index *(kg/m*^*2*^*)*	27.7 ± 5.9	28.1 ± 4.1
Smoker *(no. of patients)*	12 (32%)	9 (20%)
Radial nerve palsy *(no. of patients)*	3 (8%)	2 (5%)
Physical activity score†	2.1 ± 1.2	2.1 ± 1.3
AO/OTA classification^35^ *(no. of patients)*		
Type A (simple)	34 (89%)	36 (82%)
Type B (wedge fragment)	4 (11%)	7 (16%)
Type C (segmental)	0 (0%)	1 (2%)
Fracture location *(no. of patients)*		
Proximal	2 (5%)	5 (11%)
Mid	35 (92%)	37 (84%)
Distal	1 (3%)	2 (5%)
Injury mechanism *(no. of patients)*		
Low energy	34 (89%)	38 (86%)
High energy	4 (11%)	6 (14%)
Dominant limb injured *(no. of patients)*	20 (53%)	18 (41%)
Pre-injury 15D score‡	0.95 ± 0.05	0.94 ± 0.05
Working status *(no. of patients)*		
Employed	25 (66%)	30 (68%)
Unemployed§	13 (34%)	14 (32%)
Physical workload among employed# *(no. of patients)*		
Physically demanding work	9 (36%)	12 (40%)
Light or sedentary work	16 (64%)	18 (60%)

* Values are given as the mean ± standard deviation, with or without the range in parentheses, or as the number of patients, with the percentage in parentheses.

† Physical activity score: 1 = physically active nearly daily, 2 = 2 to 4 times per week, 3 = approximately once per week, 4 = less than once per week, 5 = no physical activities.

‡ The 15-dimensional instrument is a generic health-related quality-of-life instrument (range, 1 [full health] to 0 [death]). Values higher than 0.9 are comparable with those of a randomly selected Finnish population aged 30 years or older. At baseline, the patient was asked to report the situation just before the fracture.

§ Patients who were students, jobless, or retired were grouped as unemployed.

# Percentages are based on the total number of employed patients in the group.

### Cost Analysis

From a societal perspective, the mean total cost per patient in the surgical group was €23,680 (95% CI, €19,165 to €28,199). The bracing group had a substantially higher mean cost per patient of €30,389 (95% CI, €21,123 to €39,654). Within the bracing group, patients who required surgery during follow-up had a notably higher mean cost per patient (€49,659; 95% CI, €27,639 to €71,679) than those with uneventful healing (€23,796; 95% CI, €13,368 to €29,423). The SUR and bootstrap simulation of the base case showed that bracing was €7,419 (95% CI, €2,381 to €12,512) more expensive than surgical treatment.

When excluding productivity losses, the cost dynamics shifted: the mean cost was €10,967 (95% CI, €10,161 to €11,776) for surgical treatment compared with €4,904 (95% CI, €3,663 to €6,144) for bracing. In the SUR and bootstrap simulation, bracing was €6,091 (95% CI, €5,484 to €6,763) less expensive than surgical treatment. Work absence was a major driver of the difference between the societal and health-care perspectives, with a median of 90 WADs (interquartile range [IQR], 51 to 96) in the surgical group and a median of 129 WADs (IQR, 64 to 224) in the bracing group. Details of the direct costs and productivity losses are given in Table 2.

**Table 2:** Breakdown of Direct Medical Costs and Productivity Losses for Both Treatment Groups*

	Unit Cost	Surgery Group (N = 38)			Functional Bracing Group (N = 44)		
		Total No. of Units	Mean Cost of Unit per Patient	Total Cost of Units	Total No. of Units	Mean Cost of Unit per Patient	Total Cost of Units
Surgery							
Primary surgery	€4,417	38	€4,417	€167,846	2	€201	€8,834
Secondary surgery	€3,167	0	€0	€0	12	€862	€37,912
Anesthesia, general	€525	38	€525	€19,950	14	€167	€7,335
Anesthesia, superior trunk block	€508	38	€508	€19,304	14	€161	€7,097
Admissions							
Community hospital bed, day	€387	11	€112	€4,257	4	€35	€1,548
Central hospital bed, day	€703	0	€0	€0	6	€94	€4,157
University hospital bed, day	€878	157	€3,628	€137,846	66	€1,317	€57,948
Diagnostics							
Radiograph	€38	228	€226	€8,596	302	€259	€11,390
EMNG	€302	10	€79	€3,011	6	€41	€1,812
Other diagnostics	€192-€195	1	€5	€195	2	€9	€384
Primary care							
Emergency department visit	€307	49	€394	€14,983	53	€368	€16,209
Primary care clinic, physician visit	€95	15	€37	€1,419	36	€77	€3,393
Primary care clinic, physician call	€52	6	€8	€310	25	€29	€1,289
Primary care clinic, nurse visit	€52	12	€16	€622	8	€9	€414
Primary care clinic, nurse call	€30	0	€0	€0	4	€3	€119
Occupational physician visit	€71	1	€2	€71	1	€2	€71
Follow-up							
Specialist visit	€121	178	€560	€21,286	240	€653	€28,717
Rehabilitation							
Physiotherapy, public sector	€96	152	€383	€14,536	218	€474	€20,841
Physiotherapy, private sector	€60	0	€0	€0	4	€5	€240
Occupational therapy	€87	5	€11	€435	6	€12	€522
Medications							
Medications total			€56	€2,140		€126	€5,522
**Total without productivity loss**			€10,967	€416,807		€4,904	€215,754
Productivity loss							
WAD	€222	2,183	€12,713	€483,106	5,141	€25,485	€1,121,336
**Total**			€23,680	€899,913		€30,389	€1,337,090

* EMNG = electromyoneurography, WAD = work absence day.

### Cost-Effectiveness Analysis

**Fig. 2:**
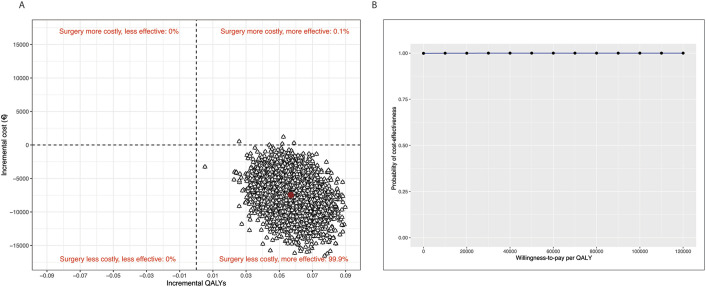
**Fig. 2-A:** Cost-effectiveness plane (base case illustrated as a red dot) displaying the distribution of bootstrapped estimates for surgical versus nonsurgical treatment, including all costs. **Fig. 2-B:** Cost-effectiveness acceptability curve (CEAC) showing the probability of surgical treatment being cost-effective at different willingness-to-pay thresholds, including all costs.

The base-case analysis from the societal perspective favored surgical treatment, with an INMB of €9,423 (95% CI, €4,139 to €14,609) at a WTP threshold of €35,000 per QALY. The cost-effectiveness plane for the base case showed that bootstrapped estimates were overwhelmingly in the lower right quadrant (99.9% of estimates), indicating that surgical treatment was both more effective and less costly than bracing (Fig. 2-A). Correspondingly, the CEAC demonstrated that surgery was almost certainly cost-effective across all WTP thresholds up to €120,000 per QALY (Fig. 2-B).

**Fig. 3:**
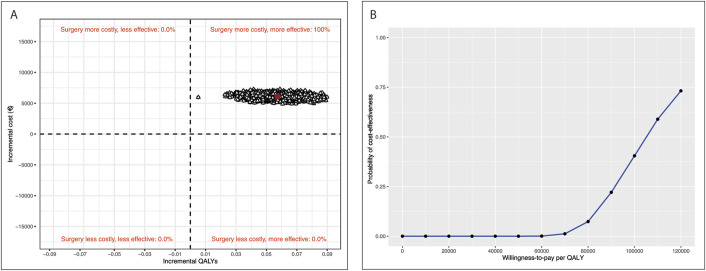
**Fig. 3-A:** Cost-effectiveness plane (base case illustrated as a red dot) displaying the distribution of bootstrapped incremental costs and QALYs for surgical versus nonsurgical treatment, considering only direct medical costs. **Fig. 3-B:** Cost-effectiveness acceptability curve (CEAC) showing the probability of surgical treatment being cost-effective at different willingness-to-pay thresholds, considering only direct medical costs.

When analyzed from the health-care perspective—i.e., excluding productivity losses—the results reversed, favoring functional bracing. In this analysis, surgery was more effective but more costly, with an INMB of −€4,087 (95% CI, −€5,215 to −€3,054). The corresponding cost-effectiveness plane showed estimates shifting to the upper right quadrant (Fig. 3-A), and the CEAC indicated that surgery was unlikely to be cost-effective at WTP thresholds below €80,000 per QALY, reaching a 75% probability of cost-effectiveness only at €120,000 (Fig. 3-B).

**Fig. 4:**
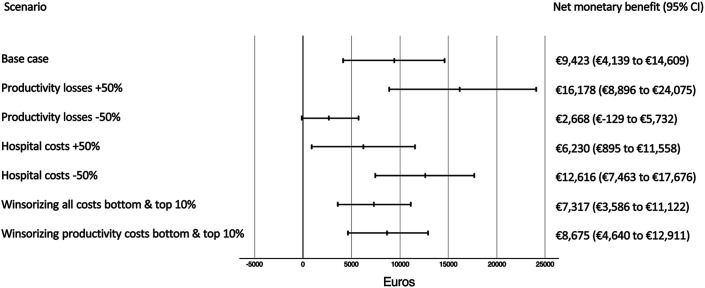
Forest plot of INMBs from 1-way sensitivity analyses, illustrating the impact of varying key cost assumptions on the cost-effectiveness results, including all costs.

The findings were robust in all 1-way sensitivity analyses (Fig. 4). The INMB increased the most when productivity losses were raised by 50%, and it remained positive, although less certain, when productivity losses were reduced by 50%. Winsorization had a minor impact, and all scenarios continued to support the cost-effectiveness of surgery.

### QALYs Using 15D

The time-weighted mean 15D contributions to QALYs from 6 weeks post-injury to the 2-year follow-up were 0.933 (95% CI, 0.910 to 0.956) in the surgical group and 0.902 (95% CI, 0.878 to 0.927) in the bracing group. These values corresponded to cumulative QALYs of 1.776 (95% CI, 1.725 to 1.827) in the surgical group and 1.705 (95% CI, 1.641 to 1.769) in the bracing group. The difference in 15D scores was 0.029 (95% CI, 0.019 to 0.043), and the difference in QALYs was 0.071 (95% CI, 0.012 to 0.130), favoring surgery.

## Discussion

### Principal Findings

This trial-based cost-effectiveness analysis found that, over the course of the initial 2-year period after humeral shaft fracture, surgical treatment was more cost-effective than bracing from a societal perspective. Although surgery involved higher initial costs, it led to faster and more reliable recovery, as previously reported, with lower total costs over 2 years^18^. Bootstrap analyses confirmed these findings, with nearly all replications showing surgery as both less costly and more effective. Across all examined WTP thresholds (€0 to €120,000), bracing was never the more cost-effective option. The main cost driver in the bracing group was the subgroup requiring secondary surgery, whose prolonged recovery led to substantially higher overall costs.

From the health-care perspective, however, bracing was the more cost-effective option due to lower direct medical costs. Surgery remained more effective but was unlikely to be cost-effective at WTP thresholds below €80,000 per QALY. The probability of surgery being cost-effective rose above 50% only at a WTP threshold around €105,000 per QALY.

### Comparison to Other Studies

Our findings contrast with those of 2 recent U.S. studies that used simulation models and secondary data^14,15^. The study by Farid et al.^14^ showed that surgery was cost-effective at 6 and 12 months at a WTP threshold of $50,000 per meaningful improvement in the Disabilities of the Arm, Shoulder and Hand (DASH) score, irrespective of productivity losses, despite primary surgery costs that were higher than those in our trial ($9,654 and €4,417, respectively). Bracing was only cost-effective if the nonunion rate was under 15.8% (or under 10.6% with productivity losses)—rates lower than typically observed. Although their overall conclusion in favor of surgery from a societal perspective aligns with our own, their $50,000 threshold per meaningful change in the DASH score (10.83 points) reflects not only a different WTP level but also a different reference for health gain compared with our €35,000 per QALY benchmark. These differences in both the valuation scale and cost threshold likely explain why operative treatment appeared more favorable in their model-based analysis than in the present trial-based evaluation^14^. In the decision-tree analysis by Fox et al.^15^, which assumed surgical costs that were nearly double those in our study, the results favored bracing at WTP thresholds of $50,000 and $100,000 per QALY, despite a 0.4 QALY gain from surgery. With productivity losses included, surgery became cost-effective only at the $100,000 threshold^15^. Additionally, a U.K.-based retrospective study found surgery to be cost-effective from a National Health Service perspective, with an ICER of £12,850 per QALY, although its assumed long-term gains in health-related quality of life are not supported by our 5-year data^16,19^.

Lastly, the recent HU-FIX (Humeral Shaft Fracture Fixation) RCT, which also compared surgery and bracing, reported results that were similar to our previously reported clinical outcomes and consistent with the present analysis, including a faster return to work with surgery compared with bracing (median, 7.3 versus 10 weeks)^17,31^.

### Strengths and Limitations

The randomized design and prospective collection of cost and outcome data support the present study’s internal validity, while real-world resource use within a centralized health-care system enhances its generalizability.

We selected the INMB rather than the ICER as the primary metric. The INMB provides clearer interpretation, avoids the instability of ratios when effect sizes are small, supports more robust statistical analysis, and has been recommended as a more intuitive and consistent framework for presenting cost-effectiveness results^32,33^.

Several limitations of our study warrant consideration. First, recall bias may have affected self-reported minor costs. However, the primary cost drivers—surgical procedures and productivity losses—were derived from objective administrative sources. The randomized design also likely balanced any residual bias.

Second, nearly three-quarters of the screened patients were not randomized, partly due to strict eligibility criteria that excluded individuals with substantial comorbidities or poor expected compliance (Fig. 1). Although these measures strengthened internal validity, they may have also introduced sampling bias, as such patients often incur higher complication-related costs. The exclusion of these patients may therefore limit the external generalizability of our findings to the broader population of patients with humeral shaft fractures.

Third, the 2-year follow-up period limits the assessment of long-term costs, such as implant removal, which has been shown to occur in approximately 7% of surgical patients^34^ (which would amount to 3 to 4 individuals in the present trial). On the basis of our cost estimates (€2,276 per patient, including 5 WADs), this would result in an additional cost of €6,828 to €9,104 in the surgical group—a modest amount relative to total costs. A substantial shift in overall cost distribution beyond the 2-year follow-up appears unlikely for 2 key reasons: (1) 96% of total costs were incurred within the first year (€2,143,331 out of €2,237,001), and (2) the QALY difference between the treatment groups diminished by the 5-year follow-up^19^.

Finally, this analysis did not account for indirect costs, such as transportation or informal caregiving. Including these costs could provide a more comprehensive picture of the economic burden and, given the faster recovery associated with surgery, would likely strengthen the case for surgery without changing the overall conclusions.

### Conclusions

Surgical treatment was more cost-effective than bracing from a societal perspective, owing to a faster recovery and reduced productivity losses. Conversely, when analyzed strictly from a health-care perspective, bracing was the more cost-effective option due to lower direct medical costs. This finding implies that in health-care systems with resource constraints, an initial nonsurgical approach—reserving surgery for nonunion or delayed healing—may be preferable. Quality of life did not differ substantially between the 2 treatments, indicating that the choice of treatment should be guided by individual circumstances and priorities. Surgical treatment might be particularly preferable for working-age individuals because it minimizes productivity losses, whereas bracing remains a viable alternative for those less impacted by time away from work or for those wishing to avoid surgical risks. These findings highlight the importance of shared decision-making tailored to patient-specific clinical scenarios, personal preferences, and life context, promoting clinically and economically optimal care.

## Appendix

Supporting material provided by the authors is posted with the online version of this article as a data supplement at jbjs.org (http://links.lww.com/JBJS/J130).

## Data Availability

A **data-sharing statement** is provided with the online version of the article (http://links.lww.com/JBJS/J131).
